# Construction of a ceRNA Network Related to Rheumatoid Arthritis

**DOI:** 10.3390/genes13040647

**Published:** 2022-04-06

**Authors:** Zhanya Huang, Nanzhen Kuang

**Affiliations:** 1 Queen Mary School, Nanchang University, Nanchang 330006, China; 4217118236@email.ncu.edu.cn; 2 Department of Immunology, Medical College of Nanchang University, Nanchang 330006, China

**Keywords:** ceRNA, RA, mTOR pathway, dopaminergic system, Wnt signaling pathway

## Abstract

(1) Background: Rheumatoid arthritis (RA) is a common systemic autoimmune disease affecting many people and has an unclear and complicated physiological mechanism. The competing endogenous RNA (ceRNA) network plays an essential role in the development and occurrence of various human physiological processes. This study aimed to construct a ceRNA network related to RA. (2) Methods: We explored the GEO database for peripheral blood mononuclear cell (PBMC) samples and then analyzed the RNA of 52 samples (without treatment) to obtain lncRNAs (DELs), miRNAs (DEMs), and mRNAs (DEGs), which can be differentially expressed with statistical significance in the progression of RA. Next, a ceRNA network was constructed, based on the DELs, DEMs, and DEGs. At the same time, the Kyoto Encyclopedia of Genes and Genomes (KEGG) and Gene Ontology (GO) analysis were used to validate the possible function of the ceRNA network. (3) Results: Through our analysis, 389 DELs, 247 DEMs, and 1081 DEGs were screened. After this, a ceRNA network was constructed for further statistical comparisons, including 16 lncRNAs, 1 miRNA, and 15 mRNAs. According to the GO and KEGG analysis, the ceRNA network was mainly enriched in the mTOR pathway, the dopaminergic system, and the Wnt signaling pathway. (4) Conclusions: The novel ceRNA network related to RA that we constructed offers novel insights into and targets for the underlying molecular mechanisms of the mTOR pathway, the dopaminergic system, and the Wnt signaling pathway (both classic and nonclassic pathways) that affect the level of the genetic regulator, which might offer novel ways to treat RA.

## 1. Introduction

Rheumatoid arthritis (RA) is a systemic autoimmune disease with chronic inflammation of the joints, synovial cell proliferation, and invasive destruction of cartilage and bone, leading to various complications. According to statistics, RA may affect about 1% of the population [1]. Many researchers have shown a variety of signaling pathways [2,3] and candidate genes [4] that are related to RA, but the underlying mechanism is still unclear. Based on this, it is of great significance to analyze the intrinsic mechanisms within RA for offering novel ideas for the treatment of RA.

Currently, many studies have explored the underlying mechanisms within RA. They have indicated that the formation of RA stems from the complex and extensive signal transduction network of various processes, including the disordered function of the autoimmune response, inflammation, and tumor-like cell changes [5]. Moreover, the development of cutting-edge technology and the study of this complex network have enabled a transition from the macroscale, i.e., the macromolecules of biology, to the microscale, i.e., the gene level [6,7]. However, as RA is an autoimmune disease, the related studies are still mostly focused on inflammatory factors [8,9,10], such as interleukin-1 (IL-1), interleukin-6 (IL-6), tumor necrosis factor-α (TNF-α), and granulocyte-macrophage colony stimulating factor (GM-CSF), and pathways to explore the possibility of alleviating RA [11]. Therefore, we thought the construction of a ceRNA network, the network linking the genetic factors and signaling pathways, could be a novel direction.

At the genetic level, the use of noncoding RNAs (ncRNAs) to alleviate RA is a research hotspot [12,13,14]. To be specific, noncoding RNAs (ncRNA) are RNAs that lack the ability to translate into proteins and can be further divided into miRNAs, lncRNAs, and circle RNAs [15], which regulate the expression of mRNA at the level of both transcription and post-transcription [16]. Among them, lncRNAs and miRNAs have been studied more widely. For miRNA, Xu et al. found that exosome-encapsulated miR-6089 interferes with an inflammatory response in RA through targeting the TLR4 included in the signaling pathways of TLRs/NF-κB [17]. Meanwhile, the viability, proliferation, apoptosis, and migration of fibroblast-like synoviocytes (FLS) were found to be regulated by miR-338-5p within RA via targeting NFAT5 [18].

For lncRNA, Zhang et al. documented that the lncRNA HOTAIR can target downstream miR-138 to inhibit the activation of the NF-κB pathway in LPS-treated chondrocytes, which could alleviate the progression of RA, which indicates the importance of lncRNA–miRNA interactions in RA pathogenesis [19]. Furthermore, some evidence has suggested that the function of ncRNA can be more comprehensively discussed within the ceRNA network through an lncRNA–miRNA–mRNA axis within autoimmune diseases [20,21,22,23,24]. According to this principle, Zhang et al. found that the overexpression of the lncRNA ENST00000494760 may sponge up miR-654-5p, promoting the expression of C1QC in RA patients. This novel ceRNA axis can be used as a biomarker [25]. Yang et al. found that CIRCRNA_09505 can act as a miR-6089 sponge to interfere with inflammation through the miR-6089/AKT1/NF-κB axis in CIA mice (an animal model of RA) [26]. Therefore, constructing an RA-related ceRNA network based on the lncRNA–miRNA–mRNA axis has great potential significance for RA research. We assumed that an analysis of the related ceRNA network could provide novel targets for treating RA.

To construct the ceRNA network, we downloaded the microarray data of the lncRNAs, miRNAs, and mRNAs of PBMC samples (GSE101193 and GSE124373). We first screened the DELs, DEMs, and DEGs in these two datasets through GEO2R analysis. We then used the ggalluvial R package to construct lncRNA–miRNA–mRNA triplets with miRcode, miRDB, miRTarBase, and TargetScan based on DELs, DEMs, and DEGs. Finally, the Kyoto Encyclopedia of Genes and Genomes (KEGG) pathway and Gene Ontology (GO) analysis were used by the clusterProfiler R package to explore the possible functions of the ceRNA network. 

This study discriminated among human RA-related lncRNA, miRNAs, mRNAs, and possible signaling pathways with high statistical significance, which might offer a novel approach to identify pathological mechanisms and potential targets for RA.

## 2. Materials and Methods

### 2.1. Data Download

Firstly, we searched the GEO database (Gene Expression Omnibus (https://www.ncbi.nlm.nih.gov/geoprofiles, accessed on 1 January 2020) for datasets related to rheumatoid arthritis (RA) by using the keywords “rheumatoid arthritis” and “peripheral blood mononuclear cells”. Next, we searched databases that focused on comparing the genetic factors within PBMCs between the RA and control groups that had relatively sufficient samples from humans. Therefore, GSE101193 and GSE124373 were downloaded. For lncRNA expression profiling, 27 PBMC samples from RA patients and 27 PBMC samples from the healthy control were included in the GSE101193 dataset (platform: GPL21827 Agilent-079487 Arraystar Human LncRNA microarray V4). For miRNA expression profiling, 28 PBMC samples from RA patients and 18 PBMC samples from a healthy control were included in the GSE124373 dataset (platform: GPL21572 Affymetrix Multispecies miRNA-4 Array). For gene/mRNA expression profiling, we used the GSE101193 dataset.

### 2.2. DELs/DEMs/DEGs Screening

This study used GEO2R, a software platform that automatically performs deviation control analysis for differential expression analysis. Firstly, the differentially expressed lncRNAs (DELs, adj.P.Val < 0.05 and |log FC| > 1.5) between RA and normal samples were screened. At the same time, differentially expressed miRNAs (DEMs) between RA and normal samples were screened, with the cutoff criteria of a *p*-value of < 0.05. In addition, differentially expressed mRNAs (DEGs) between RA and normal samples were screened based on adj.P.Val < 0.05 and |log FC| > 1.5. Next, the DELs, DEMs, and DEGs were used for subsequent analysis.

### 2.3. CeRNA Network Construction

We used the ggalluvial R package to construct lncRNA–miRNA–mRNA triplets with miRcode (Version 11; http://www.mircode.org/mircode/, accessed on 1 January 2020), miRDB (Version 7.0; http://mirdb.org/, accessed on 1 January 2020), miRTarBase (http://mirtarbase.mbc.nctu.edu.tw/index.html, accessed on 1 January 2020) and TargetScan (Version 7.2; http://targetscan.org/vert_72/, accessed on 1 January 2020) from the DELs, DEMs and DEGs. 

MiRcode provides miRNA target predictions of the entire human genome, including more than 10,000 lncRNAs. miRDB can provide miRNA targets and functional annotations in the human genome [27,28]. TargetScan can predict miRNA binding sites, and it is very effective in predicting miRNA binding sites in mammals. MiRTarBase specializes in collecting miRNA–mRNA targeting relationships supported by experimental evidence. All databases have sufficient experimental and computational support and are similar in function but different in propensity, so their combined use can improve the quality of research.

Firstly, we predicted the miRNA targeted by the DELs and constructed the lncRNA–miRNA pairs with the miRcode database based on DELs and DEMs. Next, the target genes of these miRNA signatures were acquired using the miRDB, miRTarBase, and TargetScan databases. Genes that existed in all three databases were treated as target genes of these miRNAs. Finally, through a comparison of predicted target genes with essential genes consisting of DEGs, only the remaining overlapped genes and their interaction pairs were used to construct the lncRNA–miRNA–mRNA triplets (the ceRNA network).

### 2.4. GO and KEGG Enrichment Analysis of the ceRNA Network

**Figure 1:**
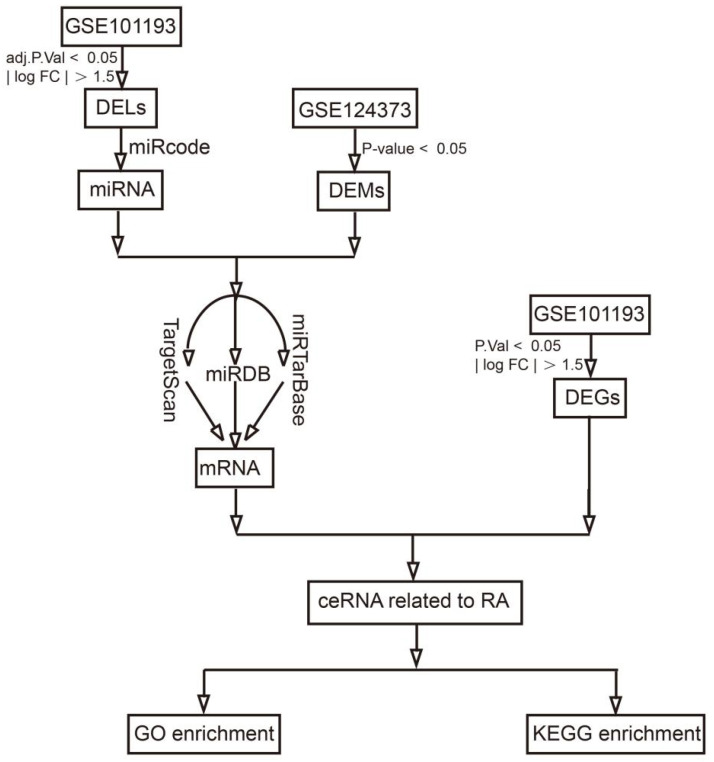
Workflow of this study.

In order to explore the possible functions of the ceRNA network, Kyoto Encyclopedia of Genes and Genomes (KEGG) pathway and Gene Ontology (GO) analyses were performed by the clusterProfiler R package. For GO analysis, a *p*-value of < 0.05 indicates statistical significance, and the GO analysis involved three categories, namely molecular function (MF), biological processes (BP), and cellular components (CC). For KEGG analysis, a *p*-value of < 0.05 was used as the cutoff criterion. The workflow of this study is shown in Figure 1.

## 3. Results

### 3.1. DELs/DEMs/DEGs

**Figure 2:**
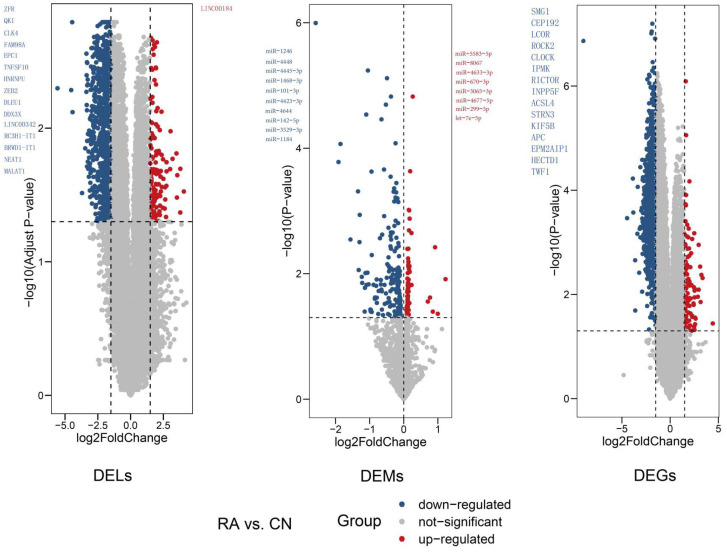
DELs/DEMs/DEGs screening.

As we know, lncRNA–miRNA pairs and miRNA–mRNA pairs can form lncRNA–miRNA–mRNA triplets. miRNA can bind to a targeted mRNA to promote mRNA degradation, while an lncRNA can bind to a targeted miRNA to inhibit mRNA degradation. The data were analyzed separately. As shown in Figure 2, 389 DELs (52 upregulated and 337 downregulated) were screened in GSE101193, 247 DEMs (71 upregulated and 176 downregulated) in GSE124373, and 1081 DEGs (97 upregulated and 984 downregulated) were screened in GSE101193. These DELs, DEMs, and DEGs were selected for subsequent analysis.

### 3.2. The ceRNA Network

**Figure 3:**
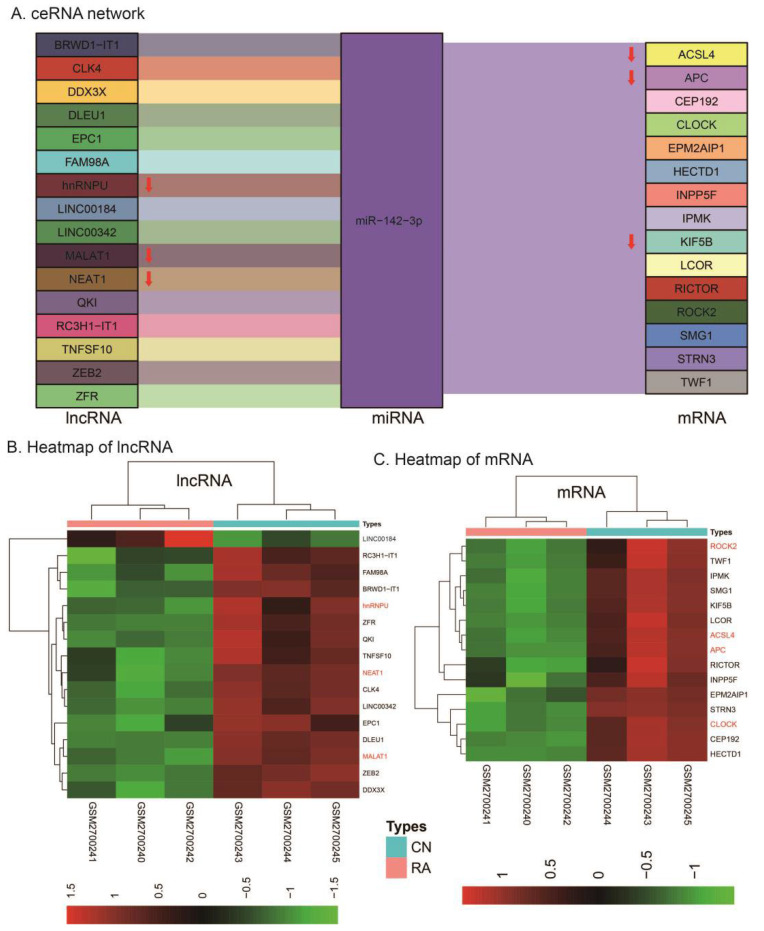
(**A**) The ceRNA network. (**B**) Heatmap of lncRNAs in the ceRNA network. (**C**) Heatmap of mRNAs in the ceRNA network.

As shown in Figure 3A, a ceRNA network, including 16 lncRNAs (especially for hnRNPU, MALAT1, and NEAT1), 1 miRNA (miR-142-3p), and 15 mRNAs (especially for ACSL4, APC, CLOCK, and ROCK), was constructed with *p*-values smaller than 0.05. Fifteen of the lncRNAs were downregulated and all 15 mRNAs were downregulated in RA (Figure 3B,C).

### 3.3. GO and KEGG Enrichment Analysis of the ceRNA Network

**Figure 4:**
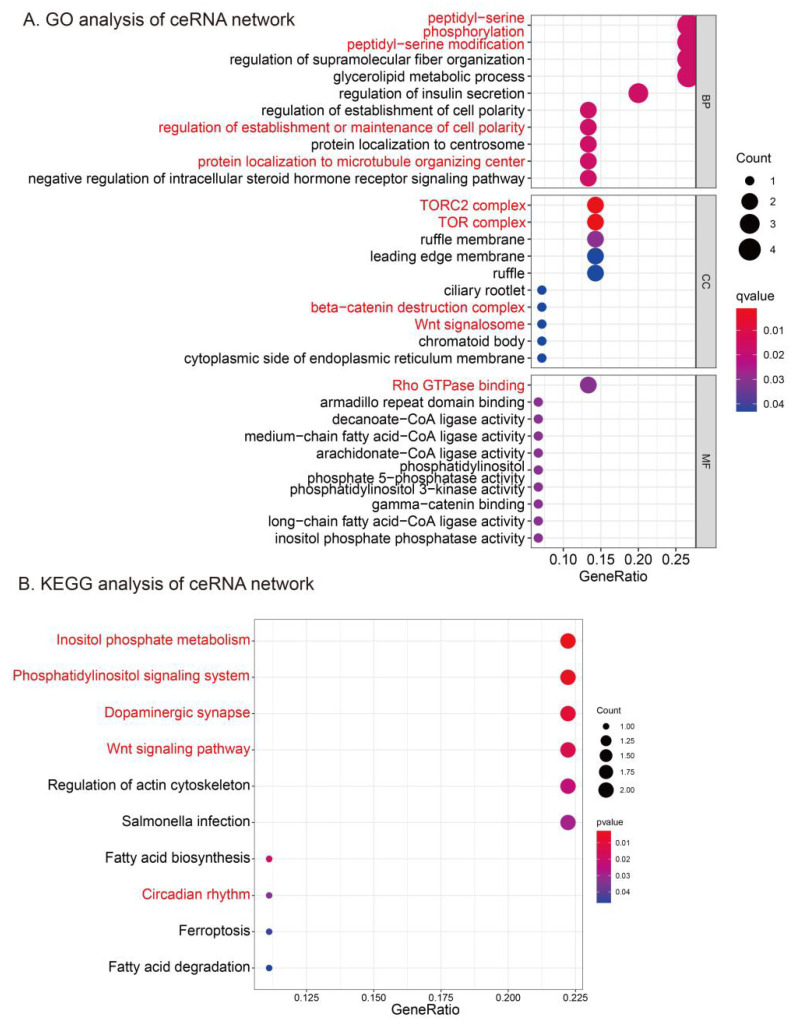
(**A**) GO and (**B**) KEGG enrichment analyses of the ceRNA network.

A GO functional annotation analysis was carried out further to test the underlying biological functions of the ceRNA network. We identified 156 significant GO-BP terms, 14 GO-CC terms, and 38 GO-MF terms (Table 1) with a *p*-value of < 0.05. The top 15 significant GO terms are shown in Figure 4A. For the GO-BP analysis of the ceRNA network, the Wnt signaling pathway (peptidyl–serine, phosphorylation, peptidyl–serine modification, protein localization to the centrosome, protein localization to the microtubule organizing center) showed significance in RA. In GO-CC analysis, the most enriched terms indicated the significance of the mTOR pathway (TORC2 complex, TOR complex) and the canonical Wnt signaling pathway (β-catenin destruction complex, Wnt signalosome). Meanwhile, the Wnt signaling pathway, especially for nonclassic pathways (Rho GTPase binding), had significance in RA according to the GO-MF terms. In addition, as exhibited in Figure 4B, the KEGG pathway enrichment analysis of the ceRNA network indicated that they were predominately enriched in 10 KEGG pathways (Table 2) based on a *p*-value of < 0.05. The dopaminergic system (dopaminergic synapse, circadian rhythm) and the Wnt signaling pathway (inositol phosphate metabolism, phosphatidylinositol signaling system, Wnt signaling pathway) were dominant.

## 4. Discussion

This study constructed an RA-related ceRNA network, screened out the factors related to RA at the gene level as comprehensively as possible, and further inferred the possible pathways from the influence of related genes on RA by GO and KEGG analysis. Mounting experiments have shown that mistakenly expressed ncRNAs, such as lncRNAs and miRNAs, may be dominant contributors to RA’s pathogenesis and progression [19,20,21,22,23,24]. Moreover, according to the ceRNA theory [29], accumulating evidence has also showed that ceRNA networks participate in regulating the viability, proliferation, migration, and apoptosis of fibroblast-like synoviocytes (FLS) within RA [30], providing novel ideas for the clinical treatment of RA progression. For instance, the lncRNA MEG3 can alleviate RA through miR-141 and inactivation of the AKT/mTOR signaling pathway [13]. The lncRNA HOTAIR can alleviate the progression of RA by targeting miR-138 and inhibiting the NF-κB pathway [19]. The lncRNA GAS5 can alleviate RA by regulating the miR-222-3p/Sirt1 signaling axis [31]. Therefore, this study might provide new guidance for the treatment of RA.

However, given that bioinformatics is a relatively new concept in the field of RA, the sample size for gene comparisons is insufficient, which may have resulted in certain false positive or false negative results. On this basis, we found that in our constructed ceRNA network, three lncRNAs (hnRNPU, MALAT1, and NEAT1), one miRNA (miR-142-3p), and four mRNAs (ACSL4, APC, CLOCK, and ROCK) were directly associated with RA [32,33,34,35,36,37,38,39,40,41,42,43,44,45,46]. In addition, six lncRNAs (QKI, EPC1, TNFSF10, DDX3X, RC3H1-IT1, and BRWD1-IT1) and six mRNAs (SMG1, LCOR, IPMK, RICTOR, KIF5B, and HECTD1) were confirmed to play a role in the destruction of cartilage or the promotion of inflammation [47,48,49,50,51,52,53,54,55,56,57,58], which indirectly supports their association with RA. These pieces of evidence are compatible with the findings of this research to a certain extent. Moreover, additional novel genes screened in this article (lncRNAs: ZFR, CLK4, FAM98A, ZEB2, DLEU1, LINC00184, and LINC00342; mRNAs: CEP192, INPP5F, STRN3, EPM2AIP1, and TWF1) might provide new targets for treating RA.

Further, we analyzed the downstream pathways of the ceRNA network by GO and KEGG analysis, and found that the mTOR pathway, the dopaminergic system, and the Wnt signaling pathway may play important roles in RA. On this basis, we also explored the significance of these pathways in existing studies. To be specific, for the mTOR pathway, which has with prominent statistical significance in Figure 4, Kun Chen carried out a study that showed that metformin arrests the G2/M cell cycle of FLS by downregulating the IGF-IR/PI3K/AKT/mTOR pathway, thereby inhibiting the proliferation of FLS and alleviating the progression of RA [59]. In addition, a study has shown that moxibustion can also produce similar effects by inhibiting the mTOR pathway [60]. Moreover, artesunate can alleviate the progression of RA by downregulating the PI3K/AKT/mTOR pathway to inhibit chondrocyte proliferation and accelerate FLS apoptosis and autophagy [61]. This evidence indicates the significance of the mTOR pathway in RA. However, the exciting finding is that the upstream TLR4-MyD88-MAPK signaling and the downstream NF-κB pathway of the mTOR signaling pathway [62] have been regarded as target pathways for treating RA in many studies [63,64]. Most of these articles paid more attention to whether a particular drug could modify the target signaling pathway to decrease the abnormal production of pro-inflammatory cytokines and alleviate RA instead of studying the pathways that might be influenced, such as the mTOR pathway. In this case, the mTOR pathway might play an underlying role in of how MAPK signaling and the NF-κB pathway can slow down the progression of RA. Clinically, Bruyn et al. found that the combination of everolimus (mTOR inhibitor) and trexate (MTX) was better than MTX alone, possibly due to the enhanced inhibition of the mTOR pathway [65,66]. However, treatment with mTOR blockers may have unnecessary pro-inflammatory side effects, such as increased levels of inflammatory markers in RA patients treated with everolimus [65]. Therefore, our screening of targets in this pathway may provide guidance for reducing side effects and clues for precision diagnosis and treatment.

For the dopaminergic system (Figure 4), potential dopamine functions in RA have been widely considered in recent decades [67]. Dopamine can indirectly affect the immune system through prolactin [68,69,70] or can directly affect immune cells through the dopamine receptors (DR) expressed by immune cells [71]. The effects of dopamine are exerted on the basis of the dose-dependent differences and different states (activated and nonactivated) of cells [67], resulting in the different roles of dopamine in the physiologic and pathologic environment. In general, dopamine is believed to inhibit the production of prolactin by stimulating D2-like DR, thus treating RA. Based on a comparison between RA patients and the control group, it was found that the number of D2DR+ B cells in the synovial tissue of RA patients is higher [72] and the number of D3DR+ mast cells is negatively correlated with the progression of the disease [73]. In blood, the number of D2DR+ B cells is positively correlated with the level of TNF in RA, suggesting that D2DR+ B cells are also involved in the systemic inflammatory response [72]. These suggest a link between dopamine and RA, but the experimental results based on this have been inconsistent or even contradictory when it comes to drug therapy. Studies have been conducted on cabergoline, a D2-like agonist, by Mobini et al. [74] and Erb et al. [75]; bromocriptine, a D2-like agonist, by McMurray [76] and Figueroa et al. [77]; and quinagolide, a D2-like agonist, by Eijsbouts et al. [78]. The different results in these experiments are likely due to the universality of the drug’s effects; i.e., the effects of the drug on RA do not necessarily affect the dopamine system alone. Therefore, the current experimental verification cannot accurately explain the specific connection between dopamine and RA. Clinically, abatacept (CTLA-4Ig), a biologic commonly used in RA patients, was found to be dependent on the Wnt pathway by Rosser-Page et al. [79,80]. However, prudent treatment should be exercised in patients with immune insufficiency, otherwise unexpected bone formation may result from a lack of T cells or Wnt-10b [79]. Considering precision medicine at the genetic level has a chance to ameliorate this side effect, target screening based on this pathway has certain significance. In addition, through a clinical trial, Briot et al. found that two commonly used RA treatment drugs anakinra (IL-1 receptor antagonist) and tocilizumab (anti-IL-6 monoclonal antibody) might also depend on the Wnt pathway to function [81]. In this study, we found that two mRNAs, CLOCK and KIF5B, related to RA can regulate the dopaminergic system so that by interfering with these two mRNAs, researchers can more precisely explore the mechanism of action between dopamine and RA, which might provide novel ideas for treating RA.

For the Wnt signaling pathway, RA treatment through the canonical Wnt/β-catenin pathway has been primarily described [82]. Xiao Wang et al. found that capsules of the traditional Chinese medicine compound huangqin qingre chubi may alleviate the progression of RA by inhibiting the CUL4B/Wnt pathway [83]. However, in this study (Figure 4), the protein functions related to noncanonical signaling pathways (protein localization to the microtubule organizing center, rho GTPase binding, the TORC2 complex, the TOR complex, the phosphatidylinositol signaling system) showed more considerable statistical significance than the protein functions related to the canonical signaling pathways (β-catenin destruction complex), which suggests that the noncanonical signaling pathways may be even more critical for RA than canonical signaling pathways, or at least as necessary. This study also indicated the upstream regulators (miRNA and lncRNA) of the Wnt signaling pathway (Figure 3), which might be used as novel targets for treating RA. Among these, the only screened miRNA, miR-142-3p, may be of great research value. It has been shown that upregulation of miR-142-3p alters the effects of the NF-κB pathway and plays a role in the progression of RA [84]. In addition, the NF-κB pathway has been shown to interact with the Wnt pathway to mediate inflammatory responses [85]. Therefore, we suggest that the relationship between these two pathways and miR-142-3p is worthy of further study. Clinically, drugs used to treat RA through the dopamine system mainly focus on cabergoline and bromocriptine [74,75,86,87]. However, clinical evidence in recent years has found that the regulation of the dopamine pathway seems to regulate the progression of RA to a certain extent, but there is no definite treatment mechanism [69]. Therefore, further analysis from the genetic perspective is meaningful.

This study has some limitations because of the lack of experimental verification. Moreover, DEMs and DEGs were screened on the basis of a *p*-value smaller than 0.05 instead of an adj.P.Value smaller than 0.05 because if adj.P.Val < 0.05 were used as the screening condition, the DEMs and DEGs that can be screened are very few, which would have been insufficient for constructing a ceRNA network. However, this does not necessarily mean that the DEMs and DEGs screened in this study do not have sufficient significance. In fact, when the screening conditions are very strict, the probability of false negatives occurring will also increase. Therefore, we appropriately increased the scope of screening, and discussed the screened miRNA and mRNA in line with previous experiments [59,60,61,62,63,64,65,66,67,68,69,70,71,72,73,74,75,76,77,78,79,80,81,82] and found that they have a certain physiological significance. Overall, this study illustrated the significant and novel factors from the gene level to the protein level, which may be regarded as experimental targets for treating RA. Furthermore, it described the possible pathways, which may suggest potential experiments on the corresponding genes and proteins. Hence, this research is of great significance for the design of experiments and the better treatment of RA. 

## 5. Conclusions

Our study used public databases to systematically analyze mRNA-miRNA–lncRNA expression profiles related to RA. In total, 16 lncRNAs (especially for hnRNPU, MALAT1, and NEAT1), 1 miRNA (miR-142-3p), and 15 mRNAs (especially for ACSL4, APC, CLOCK, and ROCK) were identified as being involved in the RA PBMC samples, which may imply three RA-related pathways including the mTOR pathway, the dopaminergic system, and the Wnt signaling pathway (both classic pathways and nonclassic pathways). On this basis, the possibility of treating RA based on the ceRNA network and related pathways was discussed. Therefore, our study might provide novel targets for treating RA.

## Figures and Tables

**Table 1:** GO enrichment analysis of the ceRNA network.

Ontology	ID	Description	BgRatio	*p*-Value	P. Adjust	Q-Value	Gene ID	Count
BP	GO:0018105	peptidyl–serine phosphorylation	310/18,866	0.0000846	0.027603925	0.016408959	SMG1/ROCK2/RICTOR/INPP5F	4
BP	GO:0018209	peptidyl–serine modification	333/18,866	0.000111566	0.027603925	0.016408959	SMG1/ROCK2/RICTOR/INPP5F	4
BP	GO:2000114	regulation of the establishment of cell polarity	22/18,866	0.000135053	0.027603925	0.016408959	ROCK2/RICTOR	2
BP	GO:1902903	regulation of supramolecular fiber organization	373/18,866	0.000172687	0.027603925	0.016408959	ROCK2/RICTOR/APC/TWF1	4
BP	GO:0032878	regulation of the establishment or maintenance of cell polarity	25/18,866	0.000175152	0.027603925	0.016408959	ROCK2/RICTOR	2
BP	GO:0071539	protein localization to the centrosome	31/18,866	0.000270738	0.027724176	0.016480441	CEP192/APC	2
BP	GO:1905508	protein localization to the microtubule organizing center	33/18,866	0.000307137	0.027724176	0.016480441	CEP192/APC	2
BP	GO:0046486	glycerolipid metabolic process	434/18,866	0.000308239	0.027724176	0.016480441	SMG1/IPMK/INPP5F/ACSL4	4
BP	GO:0033144	negative regulation of the intracellular steroid hormone receptor signaling pathway	35/18,866	0.000345793	0.027724176	0.016480441	CLOCK/STRN3	2
BP	GO:0050796	regulation of insulin secretion	181/18,866	0.00036301	0.027724176	0.016480441	CLOCK/ACSL4/KIF5B	3
BP	GO:0046488	phosphatidylinositol metabolic process	185/18,866	0.000387013	0.027724176	0.016480441	SMG1/IPMK/INPP5F	3
BP	GO:0030073	insulin secretion	213/18,866	0.000584074	0.035403862	0.021045576	CLOCK/ACSL4/KIF5B	3
BP	GO:0090276	regulation of peptide hormone secretion	213/18,866	0.000584074	0.035403862	0.021045576	CLOCK/ACSL4/KIF5B	3
BP	GO:0072698	protein localization to the microtubule cytoskeleton	53/18,866	0.000794252	0.044560949	0.026488942	CEP192/APC	2
BP	GO:0044380	protein localization to the cytoskeleton	57/18,866	0.000918215	0.044560949	0.026488942	CEP192/APC	2
BP	GO:0046854	phosphatidylinositol phosphorylation	57/18,866	0.000918215	0.044560949	0.026488942	SMG1/IPMK	2
BP	GO:0030072	peptide hormone secretion	257/18,866	0.001007031	0.044560949	0.026488942	CLOCK/ACSL4/KIF5B	3
BP	GO:0003170	heart valve development	61/18,866	0.001050909	0.044560949	0.026488942	ROCK2/HECTD1	2
BP	GO:0046883	regulation of hormone secretion	267/18,866	0.001124321	0.044560949	0.026488942	CLOCK/ACSL4/KIF5B	3
BP	GO:0030258	lipid modification	271/18,866	0.001173562	0.044560949	0.026488942	SMG1/IPMK/INPP5F	3
BP	GO:0110053	regulation of actin filament organization	278/18,866	0.001262989	0.044560949	0.026488942	ROCK2/RICTOR/TWF1	3
BP	GO:0051298	centrosome duplication	68/18,866	0.001303984	0.044560949	0.026488942	CEP192/ROCK2	2
BP	GO:1901880	negative regulation of protein depolymerization	71/18,866	0.001420519	0.044560949	0.026488942	APC/TWF1	2
BP	GO:0000281	mitotic cytokinesis	72/18,866	0.001460434	0.044560949	0.026488942	ROCK2/APC	2
BP	GO:0046834	lipid phosphorylation	72/18,866	0.001460434	0.044560949	0.026488942	SMG1/IPMK	2
BP	GO:0032024	positive regulation of insulin secretion	74/18,866	0.001541868	0.044560949	0.026488942	ACSL4/KIF5B	2
BP	GO:0051258	protein polymerization	300/18,866	0.001571818	0.044560949	0.026488942	CEP192/RICTOR/TWF1	3
BP	GO:0033143	regulation of the intracellular steroid hormone receptor signaling pathway	75/18,866	0.001583384	0.044560949	0.026488942	CLOCK/STRN3	2
BP	GO:0070830	bicellular tight junction assembly	77/18,866	0.001668011	0.04500111	0.026750593	ROCK2/APC	2
BP	GO:0120192	tight junction assembly	79/18,866	0.00175476	0.04500111	0.026750593	ROCK2/APC	2
BP	GO:0046879	hormone secretion	314/18,866	0.001791068	0.04500111	0.026750593	CLOCK/ACSL4/KIF5B	3
BP	GO:0043242	negative regulation of protein-containing complex disassembly	81/18,866	0.001843624	0.04500111	0.026750593	APC/TWF1	2
BP	GO:0120193	tight junction organization	82/18,866	0.001888848	0.04500111	0.026750593	ROCK2/APC	2
BP	GO:0009914	hormone transport	323/18,866	0.001941672	0.04500111	0.026750593	CLOCK/ACSL4/KIF5B	3
BP	GO:0043297	apical junction assembly	85/18,866	0.002027676	0.045181911	0.026858069	ROCK2/APC	2
BP	GO:0032984	protein-containing complex disassembly	330/18,866	0.002064148	0.045181911	0.026858069	KIF5B/APC/TWF1	3
BP	GO:1901879	regulation of protein depolymerization	88/18,866	0.002171223	0.045827103	0.027241599	APC/TWF1	2
BP	GO:1903829	positive regulation of cellular protein localization	338/18,866	0.002209936	0.045827103	0.027241599	ROCK2/KIF5B/APC	3
BP	GO:0006650	glycerophospholipid metabolic process	343/18,866	0.002304246	0.046557591	0.027675832	SMG1/IPMK/INPP5F	3
BP	GO:0050708	regulation of protein secretion	352/18,866	0.00248028	0.048861513	0.029045383	CLOCK/ACSL4/KIF5B	3
BP	GO:0032956	regulation of actin cytoskeleton organization	360/18,866	0.002643621	0.050560779	0.030055499	ROCK2/RICTOR/TWF1	3
BP	GO:0090277	positive regulation of peptide hormone secretion	99/18,866	0.002737614	0.050560779	0.030055499	ACSL4/KIF5B	2
BP	GO:0061640	cytoskeleton-dependent cytokinesis	100/18,866	0.002792202	0.050560779	0.030055499	ROCK2/APC	2
BP	GO:0018108	peptidyl–tyrosine phosphorylation	374/18,866	0.00294531	0.050560779	0.030055499	RICTOR/INPP5F/TWF1	3
BP	GO:0018212	peptidyl–tyrosine modification	377/18,866	0.003012619	0.050560779	0.030055499	RICTOR/INPP5F/TWF1	3
BP	GO:0003300	cardiac muscle hypertrophy	104/18,866	0.003015681	0.050560779	0.030055499	ROCK2/INPP5F	2
BP	GO:0010923	negative regulation of phosphatase activity	104/18,866	0.003015681	0.050560779	0.030055499	CEP192/ROCK2	2
BP	GO:0002791	regulation of peptide secretion	381/18,866	0.003103842	0.050954736	0.030289684	CLOCK/ACSL4/KIF5B	3
BP	GO:0014897	striated muscle hypertrophy	107/18,866	0.00318865	0.051278702	0.030482264	ROCK2/INPP5F	2
BP	GO:0014896	muscle hypertrophy	109/18,866	0.003306505	0.052110512	0.030976727	ROCK2/INPP5F	2
BP	GO:0035305	negative regulation of dephosphorylation	111/18,866	0.003426385	0.05294101	0.031470411	CEP192/ROCK2	2
BP	GO:0051261	protein depolymerization	115/18,866	0.003672202	0.054416789	0.032347677	APC/TWF1	2
BP	GO:0032970	regulation of an actin filament-based process	405/18,866	0.003687236	0.054416789	0.032347677	ROCK2/RICTOR/TWF1	3
BP	GO:0030518	intracellular steroid hormone receptor signaling pathway	116/18,866	0.003734913	0.054416789	0.032347677	CLOCK/STRN3	2
BP	GO:0031109	microtubule polymerization or depolymerization	117/18,866	0.003798126	0.054416789	0.032347677	CEP192/APC	2
BP	GO:0042752	regulation of the circadian rhythm	122/18,866	0.004121687	0.056759572	0.033740328	ROCK2/CLOCK	2
BP	GO:0043244	regulation of protein-containing complex disassembly	122/18,866	0.004121687	0.056759572	0.033740328	APC/TWF1	2
BP	GO:0031929	TOR signaling	124/18,866	0.004254596	0.056759572	0.033740328	SMG1/RICTOR	2
BP	GO:0007098	centrosome cycle	125/18,866	0.004321795	0.056759572	0.033740328	CEP192/ROCK2	2
BP	GO:0043500	muscle adaptation	125/18,866	0.004321795	0.056759572	0.033740328	ROCK2/INPP5F	2
BP	GO:0007015	actin filament organization	434/18,866	0.004477002	0.057834057	0.034379048	ROCK2/RICTOR/TWF1	3
BP	GO:0046887	positive regulation of hormone secretion	131/18,866	0.004735354	0.060184819	0.035776442	ACSL4/KIF5B	2
BP	GO:0043434	response to peptide hormones	447/18,866	0.004862092	0.060814735	0.036150891	ROCK2/APC/EPM2AIP1	3
BP	GO:0031023	microtubule organizing center organization	136/18,866	0.005093485	0.061934727	0.036816663	CEP192/ROCK2	2
BP	GO:0006644	phospholipid metabolic process	455/18,866	0.005108829	0.061934727	0.036816663	SMG1/IPMK/INPP5F	3
BP	GO:0043401	steroid hormone-mediated signaling pathway	139/18,866	0.005314214	0.062148718	0.036943868	CLOCK/STRN3	2
BP	GO:0009306	protein secretion	462/18,866	0.005330891	0.062148718	0.036943868	CLOCK/ACSL4/KIF5B	3
BP	GO:0035592	establishment of protein localization to the extracellular region	463/18,866	0.005363087	0.062148718	0.036943868	CLOCK/ACSL4/KIF5B	3
BP	GO:0030010	establishment of cell polarity	141/18,866	0.005463792	0.062398087	0.037092104	ROCK2/RICTOR	2
BP	GO:0071692	protein localization to the extracellular region	470/18,866	0.005591787	0.062947546	0.037418726	CLOCK/ACSL4/KIF5B	3
BP	GO:0033135	regulation of peptidyl–serine phosphorylation	145/18,866	0.005768744	0.064024937	0.038059173	RICTOR/INPP5F	2
BP	GO:0007043	cell–cell junction assembly	147/18,866	0.005924107	0.064836062	0.03854134	ROCK2/APC	2
BP	GO:0016311	dephosphorylation	492/18,866	0.006348885	0.068533165	0.040739057	CEP192/ROCK2/INPP5F	3
BP	GO:1902904	negative regulation of supramolecular fiber organization	156/18,866	0.00664688	0.070780291	0.042074846	APC/TWF1	2
BP	GO:0051494	negative regulation of cytoskeleton organization	163/18,866	0.007235545	0.070827152	0.042102702	APC/TWF1	2
BP	GO:0042989	sequestering of actin monomers	10/18,866	0.007924308	0.070827152	0.042102702	TWF1	1
BP	GO:0051418	microtubule nucleation by the microtubule organizing center	10/18,866	0.007924308	0.070827152	0.042102702	CEP192	1
BP	GO:0071394	cellular response to testosterone stimulus	10/18,866	0.007924308	0.070827152	0.042102702	ROCK2	1
BP	GO:0098935	dendritic transport	10/18,866	0.007924308	0.070827152	0.042102702	KIF5B	1
BP	GO:1902946	protein localization to early endosomes	10/18,866	0.007924308	0.070827152	0.042102702	ROCK2	1
BP	GO:1904779	regulation of protein localization to centrosomes	10/18,866	0.007924308	0.070827152	0.042102702	APC	1
BP	GO:0030856	regulation of epithelial cell differentiation	171/18,866	0.007936372	0.070827152	0.042102702	ROCK2/CLOCK	2
BP	GO:0000910	cytokinesis	172/18,866	0.008026064	0.070827152	0.042102702	ROCK2/APC	2
BP	GO:0050714	positive regulation of protein secretion	172/18,866	0.008026064	0.070827152	0.042102702	ACSL4/KIF5B	2
BP	GO:0030833	regulation of actin filament polymerization	174/18,866	0.008206832	0.070827152	0.042102702	RICTOR/TWF1	2
BP	GO:0010921	regulation of phosphatase activity	175/18,866	0.008297907	0.070827152	0.042102702	CEP192/ROCK2	2
BP	GO:0007028	cytoplasm organization	11/18,866	0.008713507	0.070827152	0.042102702	KIF5B	1
BP	GO:0032253	dense core granule localization	11/18,866	0.008713507	0.070827152	0.042102702	KIF5B	1
BP	GO:0046607	positive regulation of the centrosome cycle	11/18,866	0.008713507	0.070827152	0.042102702	ROCK2	1
BP	GO:0090269	fibroblast growth factor production	11/18,866	0.008713507	0.070827152	0.042102702	ROCK2	1
BP	GO:0090270	regulation of fibroblast growth factor production	11/18,866	0.008713507	0.070827152	0.042102702	ROCK2	1
BP	GO:0099519	dense core granule cytoskeletal transport	11/18,866	0.008713507	0.070827152	0.042102702	KIF5B	1
BP	GO:1901950	dense core granule transport	11/18,866	0.008713507	0.070827152	0.042102702	KIF5B	1
BP	GO:1905245	regulation of aspartic-type peptidase activity	11/18,866	0.008713507	0.070827152	0.042102702	ROCK2	1
BP	GO:1905383	protein localization to presynapses	11/18,866	0.008713507	0.070827152	0.042102702	KIF5B	1
BP	GO:0120032	regulation of plasma membrane-bounded cell projection assembly	183/18,866	0.009042998	0.070827152	0.042102702	APC/TWF1	2
BP	GO:0060491	regulation of cell projection assembly	185/18,866	0.009233827	0.070827152	0.042102702	APC/TWF1	2
BP	GO:0031340	positive regulation of vesicle fusion	12/18,866	0.00950212	0.070827152	0.042102702	KIF5B	1
BP	GO:0032957	inositol trisphosphate metabolic process	12/18,866	0.00950212	0.070827152	0.042102702	IPMK	1
BP	GO:1905668	positive regulation of protein localization to the endosome	12/18,866	0.00950212	0.070827152	0.042102702	ROCK2	1
BP	GO:2001135	regulation of endocytic recycling	12/18,866	0.00950212	0.070827152	0.042102702	INPP5F	1
BP	GO:0008064	regulation of actin polymerization or depolymerization	190/18,866	0.009718813	0.070827152	0.042102702	RICTOR/TWF1	2
BP	GO:0070507	regulation of microtubule cytoskeleton organization	190/18,866	0.009718813	0.070827152	0.042102702	ROCK2/APC	2
BP	GO:0030832	regulation of actin filament length	191/18,866	0.009817161	0.070827152	0.042102702	RICTOR/TWF1	2
BP	GO:0002793	positive regulation of peptide secretion	193/18,866	0.010015204	0.070827152	0.042102702	ACSL4/KIF5B	2
BP	GO:0030041	actin filament polymerization	193/18,866	0.010015204	0.070827152	0.042102702	RICTOR/TWF1	2
BP	GO:0031274	positive regulation of pseudopodium assembly	13/18,866	0.010290147	0.070827152	0.042102702	APC	1
BP	GO:0032230	positive regulation of synaptic transmission, GABAergic	13/18,866	0.010290147	0.070827152	0.042102702	KIF5B	1
BP	GO:0033147	negative regulation of the intracellular estrogen receptor signaling pathway	13/18,866	0.010290147	0.070827152	0.042102702	STRN3	1
BP	GO:0042921	glucocorticoid receptor signaling pathway	13/18,866	0.010290147	0.070827152	0.042102702	CLOCK	1
BP	GO:0051988	regulation of attachment of spindle microtubules to kinetochores	13/18,866	0.010290147	0.070827152	0.042102702	APC	1
BP	GO:0099640	axo-dendritic protein transport	13/18,866	0.010290147	0.070827152	0.042102702	KIF5B	1
BP	GO:1905666	regulation of protein localization to endosomes	13/18,866	0.010290147	0.070827152	0.042102702	ROCK2	1
BP	GO:0009755	hormone-mediated signaling pathway	200/18,866	0.010722411	0.070827152	0.042102702	CLOCK/STRN3	2
BP	GO:0001921	positive regulation of receptor recycling	14/18,866	0.011077589	0.070827152	0.042102702	INPP5F	1
BP	GO:0031272	regulation of pseudopodium assembly	14/18,866	0.011077589	0.070827152	0.042102702	APC	1
BP	GO:0031958	corticosteroid receptor signaling pathway	14/18,866	0.011077589	0.070827152	0.042102702	CLOCK	1
BP	GO:0038166	angiotensin-activated signaling pathway	14/18,866	0.011077589	0.070827152	0.042102702	ROCK2	1
BP	GO:0048681	negative regulation of axon regeneration	14/18,866	0.011077589	0.070827152	0.042102702	INPP5F	1
BP	GO:0051775	response to redox state	14/18,866	0.011077589	0.070827152	0.042102702	CLOCK	1
BP	GO:0070672	response to interleukin-15	14/18,866	0.011077589	0.070827152	0.042102702	ACSL4	1
BP	GO:1905205	positive regulation of connective tissue replacement	14/18,866	0.011077589	0.070827152	0.042102702	ROCK2	1
BP	GO:0071383	cellular response to steroid hormone stimulus	206/18,866	0.011345864	0.070827152	0.042102702	CLOCK/STRN3	2
BP	GO:1902905	positive regulation of supramolecular fiber organization	208/18,866	0.011557199	0.070827152	0.042102702	ROCK2/RICTOR	2
BP	GO:0035303	regulation of dephosphorylation	209/18,866	0.011663523	0.070827152	0.042102702	CEP192/ROCK2	2
BP	GO:0045216	cell–cell junction organization	210/18,866	0.011770283	0.070827152	0.042102702	ROCK2/APC	2
BP	GO:0032252	secretory granule localization	15/18,866	0.011864447	0.070827152	0.042102702	KIF5B	1
BP	GO:0070571	negative regulation of neuron projection regeneration	15/18,866	0.011864447	0.070827152	0.042102702	INPP5F	1
BP	GO:1900037	regulation of the cellular response to hypoxia	15/18,866	0.011864447	0.070827152	0.042102702	ROCK2	1
BP	GO:1901550	regulation of endothelial cell development	15/18,866	0.011864447	0.070827152	0.042102702	ROCK2	1
BP	GO:1903140	regulation of establishment of the endothelial barrier	15/18,866	0.011864447	0.070827152	0.042102702	ROCK2	1
BP	GO:1905203	regulation of connective tissue replacement	15/18,866	0.011864447	0.070827152	0.042102702	ROCK2	1
BP	GO:0007623	circadian rhythm	218/18,866	0.01264001	0.072503172	0.043099	ROCK2/CLOCK	2
BP	GO:0031269	pseudopodium assembly	16/18,866	0.01265072	0.072503172	0.043099	APC	1
BP	GO:0042532	negative regulation of tyrosine phosphorylation of STAT protein	16/18,866	0.01265072	0.072503172	0.043099	INPP5F	1
BP	GO:0045725	positive regulation of the glycogen biosynthetic process	16/18,866	0.01265072	0.072503172	0.043099	EPM2AIP1	1
BP	GO:2000651	positive regulation of sodium ion transmembrane transporter activity	16/18,866	0.01265072	0.072503172	0.043099	KIF5B	1
BP	GO:0000075	cell cycle checkpoint	219/18,866	0.012750671	0.072503172	0.043099	CLOCK/APC	2
BP	GO:0007163	establishment or maintenance of cell polarity	220/18,866	0.012861762	0.072503172	0.043099	ROCK2/RICTOR	2
BP	GO:0002064	epithelial cell development	221/18,866	0.012973283	0.072503172	0.043099	ROCK2/CLOCK	2
BP	GO:0008154	actin polymerization or depolymerization	221/18,866	0.012973283	0.072503172	0.043099	RICTOR/TWF1	2
BP	GO:0043624	cellular protein complex disassembly	224/18,866	0.013310416	0.073527015	0.043707617	APC/TWF1	2
BP	GO:0031268	pseudopodium organization	17/18,866	0.013436409	0.073527015	0.043707617	APC	1
BP	GO:0070875	positive regulation of glycogen metabolic process	17/18,866	0.013436409	0.073527015	0.043707617	EPM2AIP1	1
BP	GO:0051495	positive regulation of cytoskeleton organization	230/18,866	0.013996188	0.076062045	0.045214547	ROCK2/RICTOR	2
BP	GO:0032271	regulation of protein polymerization	231/18,866	0.014111968	0.076165963	0.045276321	RICTOR/TWF1	2
BP	GO:0035338	long-chain fatty acyl–CoA biosynthetic process	19/18,866	0.015006037	0.080440524	0.047817303	ACSL4	1
BP	GO:0032886	regulation of microtubule-based process	240/18,866	0.015172907	0.080785476	0.048022358	ROCK2/APC	2
BP	GO:0003323	type B pancreatic cell development	20/18,866	0.015789976	0.080795463	0.048028294	CLOCK	1
BP	GO:0008090	retrograde axonal transport	20/18,866	0.015789976	0.080795463	0.048028294	KIF5B	1
BP	GO:0045019	negative regulation of a nitric oxide biosynthetic process	20/18,866	0.015789976	0.080795463	0.048028294	ROCK2	1
BP	GO:0097709	connective tissue replacement	20/18,866	0.015789976	0.080795463	0.048028294	ROCK2	1
BP	GO:1902004	positive regulation of amyloid-β formation	20/18,866	0.015789976	0.080795463	0.048028294	ROCK2	1
BP	GO:1904406	negative regulation of a nitric oxide metabolic process	20/18,866	0.015789976	0.080795463	0.048028294	ROCK2	1
BP	GO:1902307	positive regulation of sodium ion transmembrane transport	21/18,866	0.016573334	0.083716582	0.049764733	KIF5B	1
BP	GO:1904886	β-catenin destruction complex disassembly	21/18,866	0.016573334	0.083716582	0.049764733	APC	1
CC	GO:0031932	TORC2 complex	12/19,559	3.13E-05	0.001788885	0.001098438	SMG1/RICTOR	2
CC	GO:0038201	TOR complex	15/19,559	4.97E-05	0.001788885	0.001098438	SMG1/RICTOR	2
CC	GO:0032587	ruffle membrane	95/19,559	0.002045063	0.049081508	0.030137768	APC/TWF1	2
CC	GO:0031256	leading edge membrane	175/19,559	0.006749208	0.070207508	0.043109873	APC/TWF1	2
CC	GO:0001726	ruffle	179/19,559	0.007050656	0.070207508	0.043109873	APC/TWF1	2
CC	GO:0035253	ciliary rootlet	11/19,559	0.007847494	0.070207508	0.043109873	KIF5B	1
CC	GO:0030877	β-catenin destruction complex	12/19,559	0.008558059	0.070207508	0.043109873	APC	1
CC	GO:1990909	Wnt signalosome	12/19,559	0.008558059	0.070207508	0.043109873	APC	1
CC	GO:0033391	chromatoid body	13/19,559	0.009268152	0.070207508	0.043109873	CLOCK	1
CC	GO:0098554	cytoplasmic side of the endoplasmic reticulum membrane	15/19,559	0.010686922	0.070207508	0.043109873	EPM2AIP1	1
CC	GO:0044233	mitochondria-associated endoplasmic reticulum membrane	16/19,559	0.011395599	0.070207508	0.043109873	ACSL4	1
CC	GO:0036464	cytoplasmic ribonucleoprotein granule	233/19,559	0.011701251	0.070207508	0.043109873	ROCK2/CLOCK	2
CC	GO:0035770	ribonucleoprotein granule	243/19,559	0.012677658	0.070214721	0.043114302	ROCK2/CLOCK	2
CC	GO:0000242	pericentriolar material	21/19,559	0.014931918	0.076792722	0.047153426	CEP192	1
MF	GO:0017048	Rho GTPase binding	162/18,352	0.007540672	0.065516951	0.030425828	ROCK2/STRN3	2
MF	GO:0070016	armadillo repeat domain binding	10/18,352	0.008145489	0.065516951	0.030425828	STRN3	1
MF	GO:0102391	decanoate-CoA ligase activity	10/18,352	0.008145489	0.065516951	0.030425828	ACSL4	1
MF	GO:0031956	medium-chain fatty acid–CoA ligase activity	11/18,352	0.008956623	0.065516951	0.030425828	ACSL4	1
MF	GO:0047676	arachidonate-CoA ligase activity	11/18,352	0.008956623	0.065516951	0.030425828	ACSL4	1
MF	GO:0034595	phosphatidylinositol phosphate 5-phosphatase activity	12/18,352	0.009767138	0.065516951	0.030425828	INPP5F	1
MF	GO:0035004	phosphatidylinositol 3-kinase activity	12/18,352	0.009767138	0.065516951	0.030425828	IPMK	1
MF	GO:0045295	γ-catenin binding	12/18,352	0.009767138	0.065516951	0.030425828	APC	1
MF	GO:0004467	long-chain fatty acid–CoA ligase activity	13/18,352	0.010577034	0.065516951	0.030425828	ACSL4	1
MF	GO:0052745	inositol phosphate phosphatase activity	13/18,352	0.010577034	0.065516951	0.030425828	INPP5F	1
MF	GO:0019902	phosphatase binding	194/18,352	0.010663145	0.065516951	0.030425828	CEP192/STRN3	2
MF	GO:0003996	acyl-CoA ligase activity	16/18,352	0.013003014	0.065516951	0.030425828	ACSL4	1
MF	GO:0008574	ATP-dependent microtubule motor activity, plus-end-directed	16/18,352	0.013003014	0.065516951	0.030425828	KIF5B	1
MF	GO:0052744	phosphatidylinositol monophosphate phosphatase activity	18/18,352	0.014617248	0.065516951	0.030425828	INPP5F	1
MF	GO:0051010	microtubule plus-end binding	20/18,352	0.016229019	0.065516951	0.030425828	APC	1
MF	GO:0015645	fatty acid ligase activity	22/18,352	0.017838328	0.065516951	0.030425828	ACSL4	1
MF	GO:0017049	GTP-rho binding	22/18,352	0.017838328	0.065516951	0.030425828	ROCK2	1
MF	GO:0050321	tau-protein kinase activity	22/18,352	0.017838328	0.065516951	0.030425828	ROCK2	1
MF	GO:0070840	dynein complex binding	23/18,352	0.018642061	0.065516951	0.030425828	APC	1
MF	GO:0008017	microtubule binding	265/18,352	0.019269691	0.065516951	0.030425828	KIF5B/APC	2
MF	GO:0016405	CoA-ligase activity	26/18,352	0.021049578	0.068160538	0.031653501	ACSL4	1
MF	GO:0003785	actin monomer binding	28/18,352	0.022651526	0.070013807	0.032514152	TWF1	1
MF	GO:0016878	acid-thiol ligase activity	30/18,352	0.024251027	0.071232516	0.033080116	ACSL4	1
MF	GO:0051721	protein phosphatase 2A binding	32/18,352	0.025848084	0.071232516	0.033080116	STRN3	1
MF	GO:0052866	phosphatidylinositol phosphate phosphatase activity	33/18,352	0.026645697	0.071232516	0.033080116	INPP5F	1
MF	GO:1990939	ATP-dependent microtubule motor activity	34/18,352	0.027442701	0.071232516	0.033080116	KIF5B	1
MF	GO:0042162	telomeric DNA binding	36/18,352	0.029034882	0.071232516	0.033080116	SMG1	1
MF	GO:0045296	cadherin binding	332/18,352	0.029331036	0.071232516	0.033080116	KIF5B/TWF1	2
MF	GO:0016877	ligase activity, forming carbon–sulfur bonds	40/18,352	0.032211948	0.075531465	0.035076532	ACSL4	1
MF	GO:0015631	tubulin binding	365/18,352	0.034916716	0.079144557	0.036754438	KIF5B/APC	2
MF	GO:0048156	tau protein binding	45/18,352	0.036169637	0.079339848	0.036845131	ROCK2	1
MF	GO:0070888	E-box binding	50/18,352	0.040112215	0.085238458	0.039584423	CLOCK	1
MF	GO:0004402	histone acetyltransferase activity	55/18,352	0.044039738	0.085703637	0.039800451	CLOCK	1
MF	GO:0017016	Ras GTPase binding	415/18,352	0.044107496	0.085703637	0.039800451	ROCK2/STRN3	2
MF	GO:0043022	ribosome binding	57/18,352	0.045606543	0.085703637	0.039800451	RICTOR	1
MF	GO:0061733	peptide–lysine–N-acetyltransferase activity	57/18,352	0.045606543	0.085703637	0.039800451	CLOCK	1
MF	GO:0031267	small GTPase binding	428/18,352	0.046632861	0.085703637	0.039800451	ROCK2/STRN3	2
MF	GO:0004674	protein serine/threonine kinase activity	435/18,352	0.048014931	0.085921455	0.039901604	SMG1/ROCK2	2

**Table 2:** KEGG enrichment analysis of the ceRNA network.

ID	Description	BgRatio	*p*-Value	P. Adjust	Q-Value	Gene ID	Count
hsa00562	inositol phosphate metabolism	73/8104	0.002765887	0.128071623	0.109375964	IPMK/INPP5F	2
hsa04070	phosphatidylinositol signaling system	97/8104	0.004832891	0.128071623	0.109375964	IPMK/INPP5F	2
hsa04728	dopaminergic synapse	132/8104	0.008794936	0.155377197	0.132695521	CLOCK/KIF5B	2
hsa04310	Wnt signaling pathway	166/8104	0.013659929	0.180994061	0.154572882	ROCK2/APC	2
hsa00061	fatty acid biosynthesis	18/8104	0.019823143	0.202227639	0.172706822	ACSL4	1
hsa04810	regulation of the actin cytoskeleton	218/8104	0.022893695	0.202227639	0.172706822	ROCK2/APC	2
hsa05132	Salmonella infection	249/8104	0.029353631	0.222248917	0.18980543	ROCK2/KIF5B	2
hsa04710	circadian rhythm	31/8104	0.033921832	0.224732137	0.191926155	CLOCK	1
hsa04216	ferroptosis	41/8104	0.044644012	0.24791126	0.211721632	ACSL4	1
hsa00071	fatty acid degradation	43/8104	0.046775709	0.24791126	0.211721632	ACSL4	1

## Data Availability

Not applicable.
